# The limit of detection of a *Phytophthora agathidicida* LAMP assay is higher under alternative laboratory conditions

**DOI:** 10.17912/micropub.biology.002163

**Published:** 2026-09-29

**Authors:** Jade TT Palmer, Monica L Gerth

**Affiliations:** 1 School of Biological Sciences, Victoria University of Wellington, New Zealand

## Abstract

Surveillance of *Phytophthora agathidicida* , the causal agent of kauri dieback, has included soil baiting followed by loop-mediated isothermal amplification (LAMP)-based detection. This approved assay was reported to detect 1 fg of DNA using OptiGene Isothermal Master Mix on a BioRanger instrument. We evaluated its analytical robustness using the same master mix on an alternative platform (QuantStudio 3), and separately tested an alternative NEB master mix. Both assays yielded a reliable detection limit of 1,000 fg, with variable detection at 100 fg and none below. Diagnostic laboratories should verify assay performance under their operational conditions prior to implementation.

**Figure 1. Comparison of P. agathidicida LAMP assay sensitivity and reproducibility across two master mixes:** Amplification plots showing raw fluorescence over a 50-minute incubation. For each master mix, an independent 10-fold serial dilution of *P. agathidicida* genomic DNA (100,000 fg to 1 fg) was tested in technical triplicate (n=3). (A) OptiGene Isothermal Master Mix. (B) NEB WarmStart Fluorescent LAMP Master Mix. Coloured lines represent the dilution series; the black dashed line represents the no-template control (ntc).

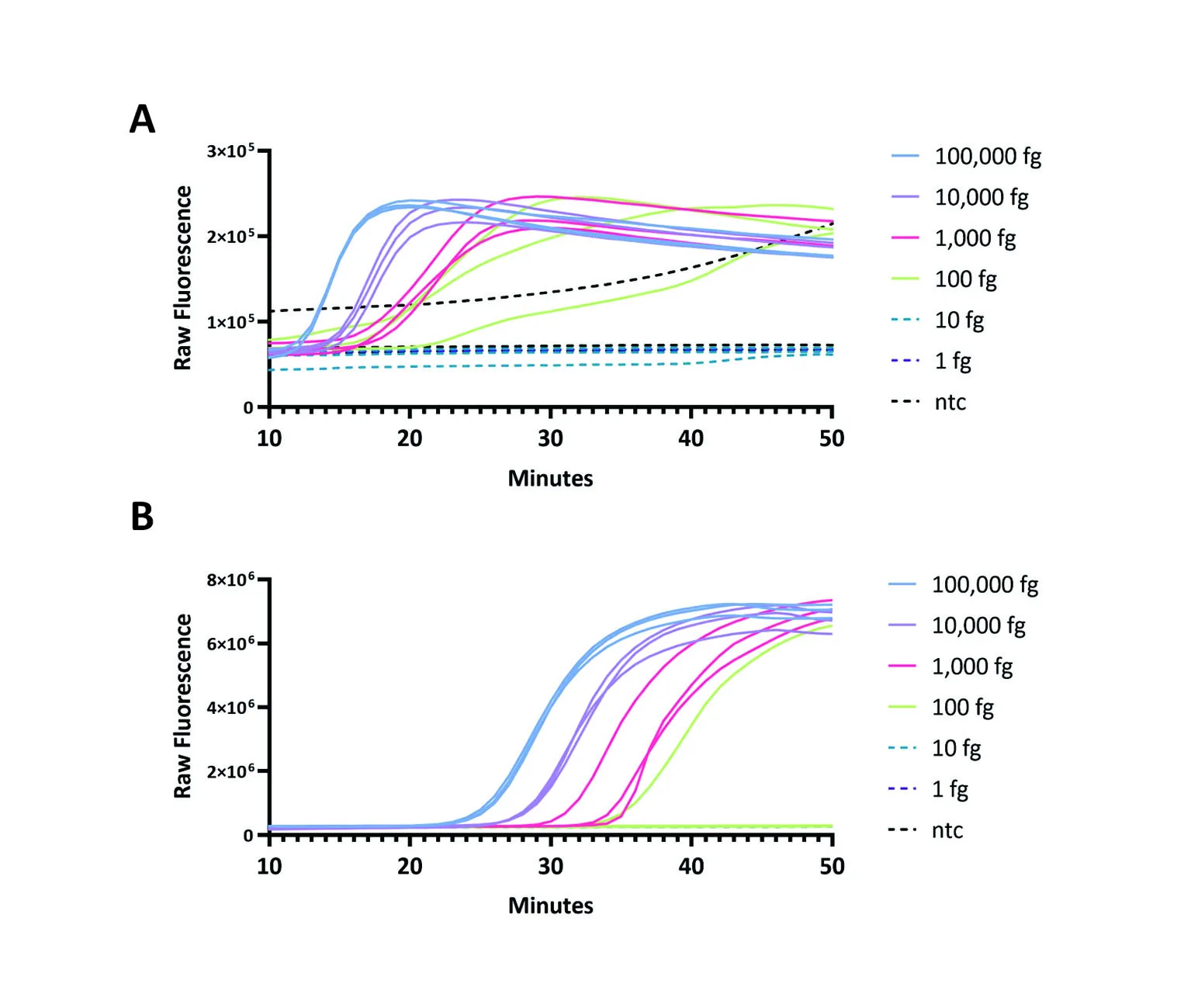



## Description

*Phytophthora agathidicida* is the causal agent of kauri dieback disease in New Zealand. Sensitive and reliable detection of this plant pathogen is vital for effective disease management. Traditional detection methods rely on soil baiting followed by morphological identification (Beever et al., 2010; Tiakina Kauri, 2023). In 2020, a hybrid baiting/loop-mediated isothermal amplification (LAMP) assay was reported that applies LAMP directly to bait material, offering a faster alternative to culturing (Winkworth et al., 2020). The original assay was optimised and validated using OptiGene Isothermal Master Mix on a dedicated LAMP instrument (BioRanger, Diagenetix Inc.), reporting a limit of detection of 1 fg of total *P. agathidicida* DNA.

We initially explored using the *P. agathidicida* LAMP primers as part of a colorimetric, field-friendly workflow designed to couple with our oospore DNA extraction method (Palmer & Gerth, 2025; Palmer et al., 2025). Initial testing yielded poor sensitivity, which was not unexpected given that colorimetric readouts can exhibit reduced sensitivity relative to fluorometric detection (Aglietti et al., 2024). To investigate further, we re-examined the baseline assay performance in the literature. While an analytical sensitivity of 1 fg was reported in the text, the published amplification plots presented data down to 2 pg (2,000 fg; Winkworth et al., 2020). To clarify performance at lower concentrations, we undertook a study to directly assess the assay's limit of detection under real-time fluorescent conditions.

We attempted to replicate the reported conditions as closely as possible, using the same master mix (OptiGene Isothermal Master Mix), reported primer concentrations (F3/B3 and FIP/BIP), and incubation temperature (63 °C) as the original study (Winkworth et al., 2020). The published protocol did not report Loop F/Loop B primer concentrations, so we used the OptiGene manufacturer-recommended 0.4 µM final concentration (OptiGene. LAMP User Guide – Assay Design & Primers. https://www.optigene.co.uk/wp-content/uploads/2012/06/OptiGene-LAMP-User-Guide-Assay-Design-Primers-1.pdf). We did not have access to a BioRanger instrument, as Diagenetix, Inc. ceased operations in 2023 (PitchBook. 2026. Diagenetix company profile. https://pitchbook.com/profiles/company/112919-32). Instead, we used a QuantStudio 3 Real-Time PCR System (Applied Biosystems). While there is precedent in the literature for using real-time PCR platforms for LAMP fluorescence detection (García-Bernalt Diego et al., 2022; Stehlíková et al., 2020), instrument hardware was a primary variable differing from the original protocol. In a second experimental condition, we trialled NEB WarmStart Fluorescent LAMP Master Mix at its recommended incubation temperature (65 °C), maintaining the 0.4 µM Loop F/Loop B primer concentration in line with standard NEB guidelines (New England Biolabs. WarmStart Multi-Purpose LAMP/RT-LAMP 2X Master Mix with UDG protocol. https://www.neb.com/en-nz/protocols/warmstart-multi-purpose-lamp-rt-lamp-2x-master-mix-with-udg-protocol-neb-m1078). Reaction parameters (including primer ratios and incubation temperatures) were not independently re-optimised for either master mix on the QuantStudio platform; assay re-optimisation could plausibly improve performance, but was outside the scope of the present study.

No amplification was observed for either master mix at 1 fg or 10 fg. At 100 fg, amplification was variable: all OptiGene replicates produced a signal, but with high variability in take-off time and curve morphology, and one of three OptiGene no-template control (NTC) replicates showed both elevated initial background fluorescence and a non-specific fluorescence increase beginning at approximately 35 minutes ( Figure 1A ). This anomaly was specific to the OptiGene mix and was not observed with the NEB mix on the same QuantStudio 3 platform; we therefore cannot exclude a chemistry-specific artefact as a contributor to the variability seen at 100 fg with the OptiGene mix, which complicates interpretation of these particular data. With the NEB mix, only one of three 100 fg replicates amplified, while NTCs remained at baseline in all three replicates ( Figure 1B ). Both master mixes yielded robust, uniform sigmoidal curves at 1,000 fg.

Overall, under the experimental conditions tested here, we established a reliable detection limit of 1,000 fg for the *P. agathidicida* LAMP assay. We acknowledge that our study does not represent a direct, same-methods replication, which would require the original BioRanger instrument and exact original protocol. Instead, it evaluates assay reproducibility and robustness (Schloss, 2018), testing whether reported performance holds across alternative platforms and/or reagents. Although this LAMP assay remains officially approved for kauri dieback surveillance (Tiakina Kauri, 2023), the commercial unavailability of the BioRanger means operationalising it today inevitably requires platform adaptation. To date, no public inter-laboratory validation data exist to support such platform substitutions — a notable gap given that formal diagnostic validation frameworks treat multi-laboratory, multi-platform transferability as an essential tier for decentralised deployment (Tier 3; Cardwell et al., 2018; Groth-Helms et al., 2023). One report (Tomscha, 2026) notes an in-house comparison by the Ministry for Primary Industries’ Plant Health and Environment Laboratory that found a TaqMan qPCR assay (Than et al., 2013) to be more sensitive than the LAMP assay, without disclosing the obtained detection limits, methods or equipment used.

While these findings should not be construed as a failure of the original protocol under its specific conditions, they suggest that the assay's sensitivity is potentially not robust to platform or reagent substitution. Laboratories intending to deploy this approved workflow on alternative instruments should independently verify and re-optimise assay performance prior to relying on it for biosecurity decision-making.

## Methods

**DNA Extraction and Quantification**

Genomic DNA was extracted from *P. agathidicida* strain NZFS 3770 mycelial mats grown in potato dextrose broth (Difco). Mats were grown until they covered approximately 50% of the surface of a 90 mm Petri dish. Harvested mats were rinsed with sterile water, blotted dry, and ground to a fine powder in liquid nitrogen. Total genomic DNA was extracted using the DNeasy Plant Mini kit (Qiagen) and quantified using the Qubit dsDNA High Sensitivity Assay Kit (Invitrogen). For each assay, a fresh 10-fold serial dilution series (100,000 fg to 1 fg) was prepared in sterile PCR-grade water.

**Primer Preparation**

The primer sequences used in this study were originally described by (Winkworth et al., 2020) for the molecular detection of *P. agathidicida* . The lyophilised primers (Macrogen, Korea) were resuspended in low-EDTA TE buffer (10 mM Tris-HCl, 0.1 mM EDTA) to a stock concentration of 100 µM and stored at -20 °C. Working concentrations are provided in the Reagents section below.

**LAMP Assay Conditions and Data Analysis**

Two reagent master mixes were evaluated: the original OptiGene Isothermal Master Mix and the WarmStart Fluorescent LAMP Kit (with UDG; NEB). Each LAMP master mix was evaluated in triplicate, including a no-template control (NTC) using sterile PCR-grade water in place of genomic DNA for every run. The originally reported OptiGene Isothermal Master Mix was used in 20 µL reactions incubated at 63 °C for 50 min. The alternative master mix, WarmStart Fluorescent LAMP Kit, was used in 25 µL reactions incubated at 65 °C for 50 min. All reactions were performed on a QuantStudio 3 Real-Time PCR System (Applied Biosystems) with fluorescence readings acquired every minute. Data were plotted and analysed using GraphPad Prism (v 11.0.0).

## Reagents

**Table:** 

**Primers**		
**Name**	**Sequence (5′ - 3′)**	**Final Concentration**
PTAF3	TTATTTGAACCAACCTCATGT	0.2 μM
PTAB3	TGTTTTACCTTGGGGACAA	0.2 μM
PTAFIP	GCTGTAGATAATCCAACTTTAAATCGTTTTGGTGTATTAATACGACCCCTAC	0.6 μM
PTABIP	CCACCCCATAGCCAATCAACAATATTTTGGGGTGCAACTGTT	0.6 μM
PTALF	TTAGTTTACATTTTACTTTTCCTTTTG	0.4 μM
PTALB	CCTATTAAAGGTATTGCAGAAAATAA	0.4 μM
**Kits and Reagents**		
**Name (Supplier)**		**Catalogue Number**
DNeasy Plant Mini kit (Qiagen)		69104
Qubit dsDNA High Sensitivity Assay Kit (Invitrogen)		Q32854
WarmStart Fluorescent LAMP Kit with UDG (New England Biolabs)		E1708
Isothermal Master Mix (OptiGene)		ISO-DR001
**Culture**		
**Name**		**Source**
*Phytophthora agathidicida* NZFS 3770		Scion*

*Also deposited in the International Collection of Microorganisms as ICMP 17027.
